# Novel *PIP5K1C* variant identified in a Chinese pedigree with lethal congenital contractural syndrome 3

**DOI:** 10.1186/s12887-024-04674-6

**Published:** 2024-03-15

**Authors:** Fang Zhang, Hongmei Guo, Xinlong Zhou, Zhengxi Deng, Qiuhong Xu, Qingming Wang, Haiming Yuan, Jianhua Luo

**Affiliations:** 1 Dongguan Maternal and Child Health Care Hospital, Dongguan, 523120 China; 2 Department of Medical Genetics, Dongguan Maternal and Child Health Care Hospital, Dongguan, 523120 China; 3 Key Laboratory for Precision Diagnosis and Treatment of Severe Infectious Diseases in Children, Dongguan, 523120 China

**Keywords:** *PIP5K1C*, Lethal congenital contractural syndrome 3, Lateral ventricles

## Abstract

**Background:**

Biallelic pathogenic variants in *PIP5K1C* (MIM #606,102) lead to lethal congenital contractural syndrome 3 (LCCS3, MIM #611,369), a rare autosomal recessive genetic disorder characterized by small gestational age, severe multiple joint contractures and muscle atrophy, early death due to respiratory failure. Currently, 5 individuals with LCCS3 were reported and 5 pathogenic variants in *PIP5K1C* were identified. Here, we reported the two fetuses in a Chinese pedigree who displayed multiple joint contractures and other congenital anomalies.

**Methods:**

Trio-based whole-exome sequencing (WES) was performed for the parents and the recent fetus to detect the genetic cause for fetus phenotype.

**Results:**

A novel variant, NM_012398.3: c.949_952dup, p.S318Ifs*28 and a previously reported variant, c.688_689del, p.G230Qfs*114 (ClinVar database) in *PIP5K1C*, were detected in the individuals, and these variants were inherited from the mother and father, respectively. We described the features of multiple joint contractures in our fetuses, including bilateral talipes equinovarus, stiffness in the limbs, extended knees, persistently closed hands and overlapping fingers, which have not been delineated detailedly in previously reported LCCS3 individuals. Furthermore, novel phenotype, bilateral dilated lateral ventricles, was revealed in one fetus.

**Conclusions:**

These findings expanded the genetic variant spectrum of *PIP5K1C* and enriched the clinical features of LCCS3, which will help with the prenatal diagnosis and genetic counseling for this family.

## Background

Lethal congenital contracture syndrome (LCCS) is a heterogenous group of congenital genetic condition. It is characterized by multiple joint contractures, polyhydramnios and reduced fetal movement, often leading to the perinatal or neonatal death. Studies have suggested that LCCS is an autosomal recessive disorder causally linked to 11 genes, including *GLE1, ERBB3, PIP5K1C, MYBPC1, DNM2, ZBTB42, CNTNAP1, ADCY6, ADGRG6, NEK9* and *GLDN*. Among them, LCCS1 (MIM #253,310), LCCS7 (MIM #607,598) and LCCS11 (MIM #617,194) have been relatively frequent reported in the literature [1–6]. However, lethal congenital contracture syndrome 3 (LCCS3 MIM #611,369), caused by biallelic pathogenic variants in *PIP5K1C* (MIM #606,102), was rarely reported.

The *PIP5K1C* gene consists of 18 exons and encodes a 668-amino acid enzyme. This protein utilizes phosphatidylinositol 4-phosphate (PI4P) as a substrate to synthesize phosphatidylinositol 4,5-phosphate (PIP2) on the cell membrane [7, 8]. PIP5K1C belongs to the PIP5K1 family of enzymes, which are classified as type I phosphatidylinositol-4-phosphate 5-kinases. This family consists of three subtypes: *PIP5K1A*, *PIP5K1B*, and *PIP5K1C*. *PIP5K1A* is predominantly expressed in skeletal muscle, while *PIP5K1B* has the highest expression in the heart. To date, only 5 individuals with LCCS3 have been reported; 5 pathogenic variants in *PIP5K1C* have been identified [9, 10, ClinVar]. Here, we reported two additional individuals in a Chinese pedigree with LCCS3 caused by pathogenic variants in *PIP5K1C*. The individuals displayed some phenotypes rarely reported before. And one novel variant in *PIP5K1C*, c.949_952dup, p.S318Ifs*28 (NM_012398.3), was identified. The results of our study expanded the genetic variant spectrum of *PIP5K1C* and enriched our understanding of the clinical characteristics of this disorder, which will be beneficial for improving the prenatal diagnosis and genetic counseling for individuals with LCCS3.

## Materials and methods

### Ethical compliance

The Ethics Committee of Dongguan Maternal and Child Health Hospital (DMCH 202,307) approved this study, and written informed consent was obtained from the legal guardian for the release of any potentially identifiable image or data contained in this paper.

### Trio-based whole-exome sequencing

Trio-based whole-exome sequencing (WES) was performed for the pedigree to screen for causal variants. Sequencing was performed with an Illumina NovaSeq 6000 (Illumina, San Diego, CA, USA). Suspected variants were verified by Sanger sequencing. The pathogenicity of the variants was interpreted according to the ACMG/AMP guidelines [11].

## Results

### Clinical report

**Fig. 1:**
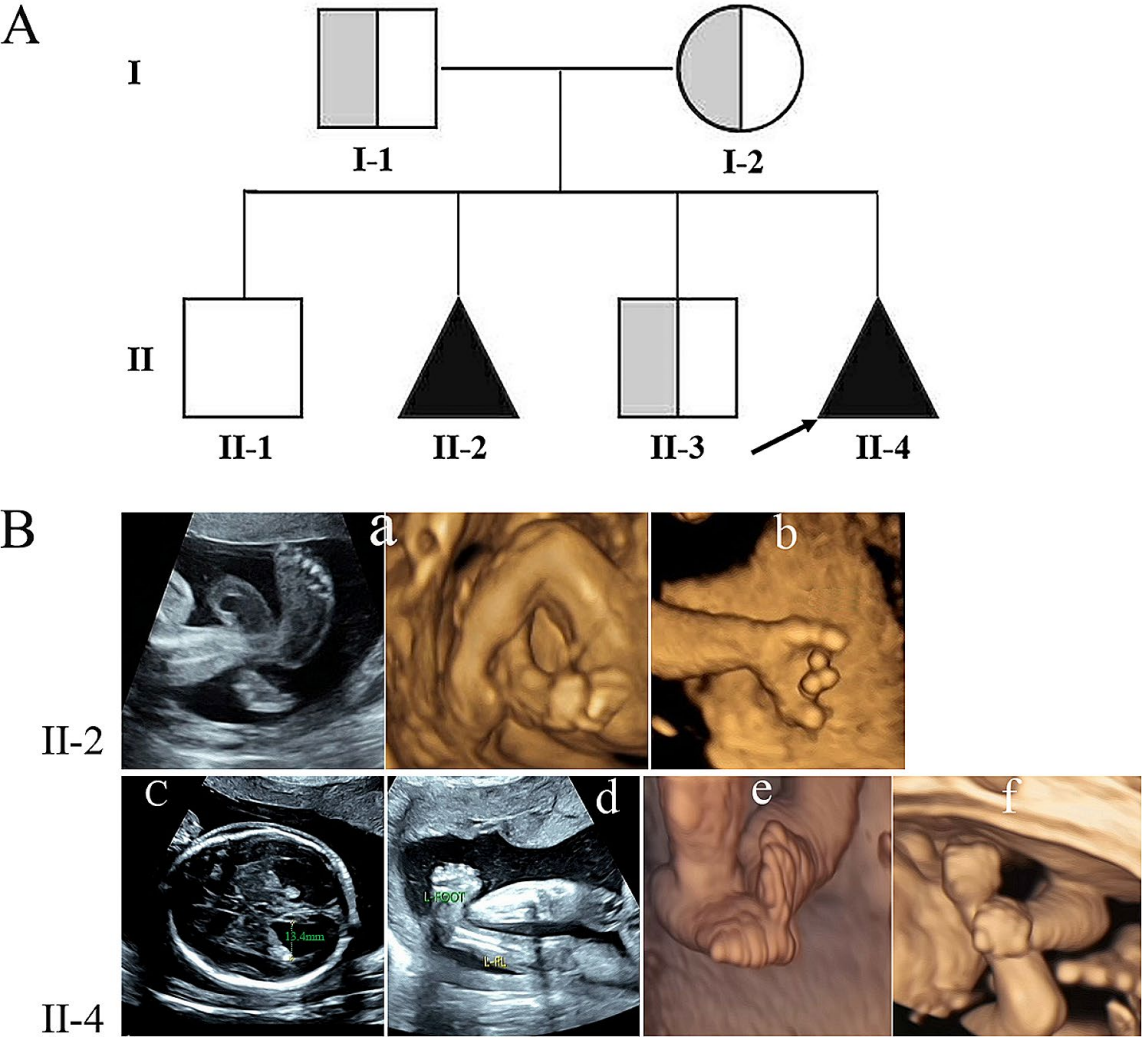
The pedigree chart and radiographic findings for the two fetuses. (**A**). Pedigree for a consanguineous Chinese family with LCCS3 (The black arrow represents the proband, translucent grey indicates carrier). (**B**). Radiographic findings for the two fetuses. (II-2) bilateral talipes equinovarus (a), flexion contractures of fingers and overlapping fingers (b). (II-4) bilateral dilated lateral ventricles (13.4 mm) (c), stiffness in the limbs, extended knees (d), bilateral talipes equinovarus (e) and persistently closed hands (f)

The two affected individuals were from a Chinese nonconsanguineous couple (Fig. 1A). The 41-year-old woman has given birth to two healthy children and experienced two eventful pregnancies. One of eventful pregnancies occurred six years ago, a prenatal ultrasound scan showed limited fetal movement, bilateral talipes equinovarus, flexion contractures of fingers and overlapping fingers at 23 weeks (Fig. 1B, II-2). Then, she underwent vaginal delivery prematurely at 26 weeks, and the baby passed away after birth due to respiratory failure. In the recent pregnancy, the woman sought medical attention due to advanced maternal age and progressively reduced fetal movement. At 23 weeks of gestation, a prenatal ultrasound scan revealed bilateral dilated lateral ventricles (13.4 mm). Additionally, the fetus exhibited stiffness in the limbs, extended knees, bilateral talipes equinovarus and persistently closed hands (Fig. 1B, II-4). The karyotype analysis and chromosomal microarray of the amniotic fluid were normal. MLPA detected no deletion of exons 7 and 8 in *SMN1*.

### Genetic analysis

**Fig. 2:**
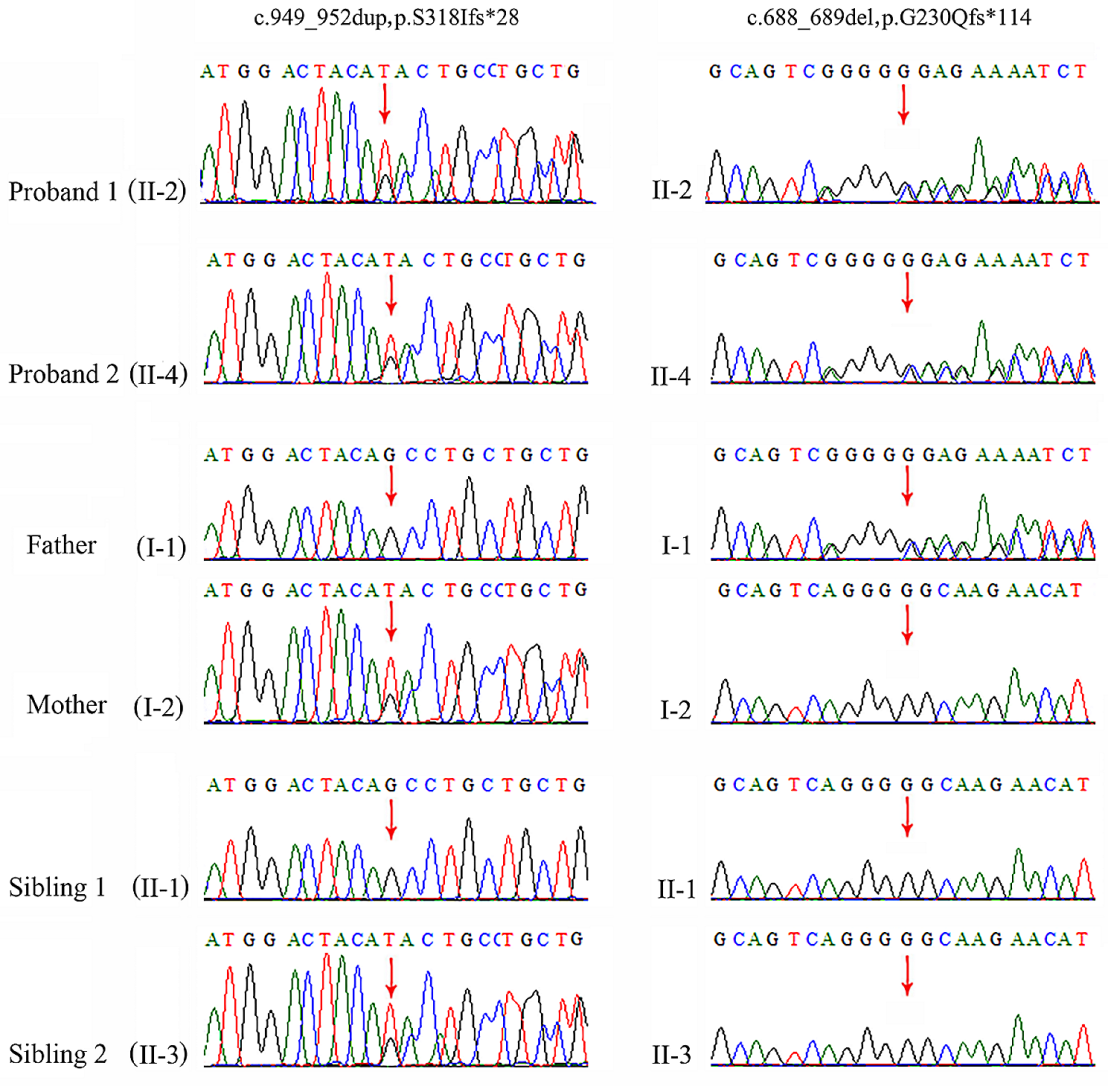
Variant confirmation by Sanger sequencing. Compound heterozygous variants NM_012398.3: c.688_689del and c.949_952dup in *PIP5K1C* were detected in both fetuses and their asymptomatic parents and siblings. The red arrow indicates the variant site

Compound heterozygous variants, NM_012398.3: c.949_952dup (p.S318Ifs*28) and c.688_689del (p.G230Qfs*114) in *PIP5K1C*, were revealed in II-4, which were inherited from the mother and father. Next, we analyzed the DNA sample of II-2, and it expectedly revealed the same *PIP5K1C* compound heterozygous variants. Sanger sequencing verification was performed for other members in the pedigree. Individual II-1 was proven to be wildtype and individual II-3 was an asymptomatic carrier (c.949_952dup, p.S318Ifs*28 in PIP5K1C). The variant segregates as autosomal recessive (Fig. 2). The paternally inherited frameshift variant, (c.688_689del, p.G230Qfs*114), has been reported in ClinVar database. The maternally inherited novel variant, c.949_952dup (p.S318Ifs*28), was predicted to cause protein truncation and was unlikely escape nonsense-mediated mRNA decay. In addition, the individuals’ phenotypes were highly consistent with that of LCCS3. Trio-based WES also excluded other possible known genetic causes. Thus, both variants were categorized as clinically pathogenic according to the ACMG/AMP guidelines. (PVS1 + PM2 + PP1 + PP4).

## Discussion

*PIP5K1C* is mainly highly expressed in brain and plays an important role in neural signaling pathway [12, 13]. *Pip5k1c -/-* mice caused a 50% reduction in PIP2 in brain, leading to an impairment of its depolarization-dependent synthesis in nerve terminals and synaptic defects [14]. *PIP5K1C* has been demonstrated to regulate various cellular processes including receptor-mediated calcium signaling transmission, actin cytoskeleton dynamics, endocytosis and exocytosis [15]. Additionally, *PIP5K1C* plays a crucial role in the maintenance of bone development. It exerts its influence on bone growth and development by regulating the movement of calcium ions in cells and body fluids [8].

**Fig. 3:**
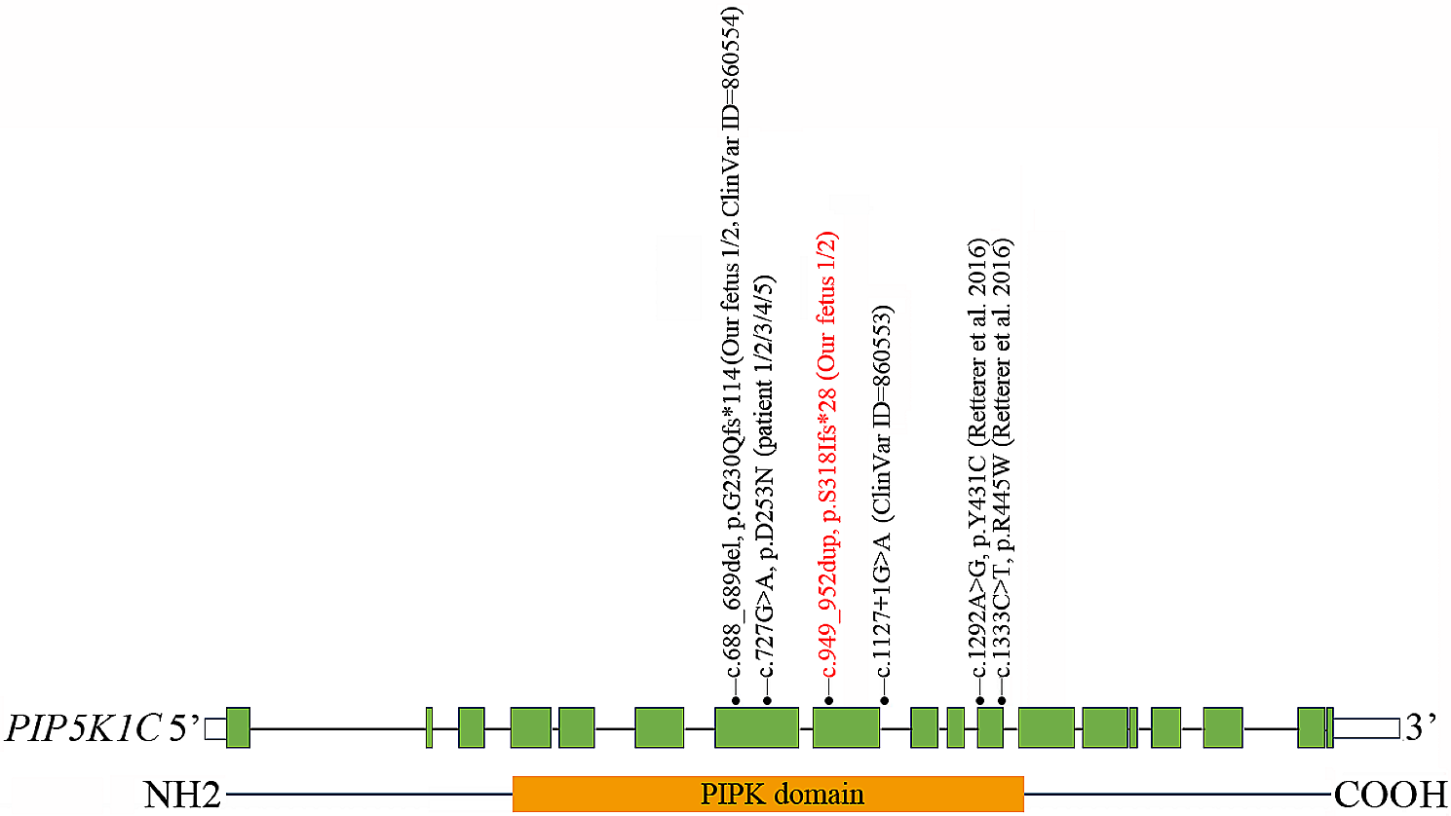
Schematic representation of *PIP5K1C* variants identified to date. The structure of *PIP5K1C* contained 18 exons (green rectangles) and introns (grey horizontal line). Lower side: the PIP5K1C protein domains: PIPK domain (76-449aa). The localization of variants and substitutions identified is depicted with dots. Black: Variants reported in the literature or ClinVar database; Red: Novel variants identified in this study

Homozygous or compound heterozygous variants in *PIP5K1C* have been known to cause LCCS3 through haploinsufficiency mechanisms. LCCS3 was a very rare and severe disorder. To date, 5 variants were identified in *PIP5K1C*. In this study, we revealed a novel variant (c.949_952dup, p.S318Ifs*28) in *PIP5K1C* (Fig. 3). The expanded mutation spectrum in *PIP5K1C* improves the molecular diagnosis of LCCS3. It was observed that all pathogenic variants in *PIP5K1C* were located in the PIPK domain (76-449aa). The variants (c.688_689del, p.G230Qfs*114 and c.727G > A, p.D253N) seem to be mutation hotspots. Certainly, it was necessary to add more clinical cases to further expand mutation spectrum.

Currently, only 7 LCCS3 individuals with detailed clinical information, including the two individuals here, have been described (Table 1). All individuals presented with dyskinesia and multiple joint contractures. Novel phenotypes, bilateral dilated lateral ventricles, were observed in our fetus 2 (II-4), which may be related to the high expression of *PIP5K1C* in the brain. However, our fetus 1 (II-2) did not show this feature. It indicates that the *PIP5K1C* variant can cause phenotypic variability even within the same family. Furthermore, it has been reported that individuals with LCCS7 or LCCS9 also displayed various brain anomalies, such as cerebral and cerebellar atrophy with almost no white matter, thin corpus callosum, and small basal ganglia and hippocampi [16, 17]. Whether brain anomaly observed in our fetus 2 is truly part of the spectrum of LCCS3 or is a coincidental finding remains to be further investigated. Although multiple joint contractures have been reported as a feature of LCCS3, the detailed phenotypes have not been displayed [9]. Here, we presented detailed presentations of multiple joint contractures in our two fetuses, including bilateral talipes equinovarus, stiffness in the limbs, extended knees, flexion contractures of fingers and overlapping fingers. Talipes equinovarus has previously been observed in individuals with LCCS9 and 10 [18, 19], here our two fetuses also exhibited bilateral talipes equinovarus. Ankylosis of knee joint was observed in individuals with LCCS6, 7 and 9, here our fetus 2 (II-4) showed this feature [5, 18, 20]. Flexion contractures of fingers were reported in individuals with LCCS7, 9, 10, 11, which was also observed in our two fetuses [5, 18–21]. Our findings profiled the picture of multiple joint contractures in LCCS3. Polyhydramnios was a marked feature of LCCS [16–25]. However, this feature has not yet been observed in individuals with LCCS3, which deserves further investigation.

**Table 1:** Overview of variants and phenotypes observed in patients with LCCS3

	Patient 1	Patient 2	Patient 3	Patient 4	Patient 5	Our fetus 1 (II-2)	Our fetus 2 (II-4)
	Narkis et al. 2007	Narkis et al. 2007	Narkis et al. 2007	Narkis et al. 2007	Narkis et al.2007		
Race	Bedouin	Bedouin	Bedouin	Bedouin	Bedouin	Chinese	Chinese
Variants	c.757G>A,	c.757G>A	c.757G>A	c.757G>A	c.757G>A	c.688_689del:	c.688_689del:
	p.Asp253Asn	p.Asp253Asn	p.Asp253Asn	p.Asp253Asn	p.Asp253Asn	p.G230Qfs*114	p.G230Qfs*114
	(homozygous)	(homozygous)	(homozygous)	(homozygous)	(homozygous)	c.949_952dup:	c.949_952dup:
						p.S318Ifs*28	p.S318Ifs*28
Clinical findings							
Dyskinesia	+	+	+	+	+	+	+
Joint contractures	+	+	+	+	+	+	+ (stiffness in the limbs, extended knees)
Hand anomalies	NA	NA	NA	NA	NA	+ (flexion contractures of fingers; overlapping fingers)	+ (persistently closed hands)
Talipes equinovarus	NA	NA	NA	NA	NA	+	+
Brain anomalies	NA	NA	NA	NA	NA	–	+ (bilateral dilated lateral ventricles)
Gestational age at birth	29 W	29 W	Full term	Full term	Full term	26 W	Termination
Outcome	Died	Died	Died	Died	Died	Died	
Cause of death	respiratory insufficiency	respiratory insufficiency	respiratory insufficiency	respiratory insufficiency	respiratory insufficiency	respiratory insufficiency	

*Abbreviation* +, present; –, absent; NA, not available

In conclusion, we described in detail the prenatal clinical features of a Chinese pedigree with LCCS3 caused by biallelic pathogenic variants in *PIP5K1C*. The identification of the novel variant and novel phenotypes expands the variant spectrum of *PIP5K1C* and enriches the clinical characteristics of LCCS3, which will be valuable for prenatal diagnosis and genetic counseling.

## Data Availability

The datasets for this article are not publicly available due to concerns regarding participant/patient anonymity. Requests to access the datasets should be directed to the corresponding author.
